# Comparison and Agreement of Echocardiographic Volumetric Methods for Quantifying Mitral Regurgitation in Dogs with Myxomatous Mitral Valve Disease

**DOI:** 10.3390/ani16081249

**Published:** 2026-04-18

**Authors:** Shimpei Kawai, Ryohei Suzuki, Yohei Mochizuki, Yunosuke Yuchi, Shuji Satomi, Arata Kitazawa, Takahiro Teshima, Hirotaka Matsumoto

**Affiliations:** 1 Laboratory of Veterinary Internal Medicine, School of Veterinary Medicine, Faculty of Veterinary Science, Nippon Veterinary and Life Science University, Musashino 180-8602, Tokyo, Japan; 2 Faculty of Veterinary Medicine, Okayama University of Science, Imabari 794-8555, Ehime, Japan

**Keywords:** diameter-based methods, echocardiography, left ventricular volume, modified Simpson’s method of discs, regurgitant fraction, regurgitant volume

## Abstract

Quantitative assessment of mitral regurgitation (MR) in dogs with myxomatous mitral valve disease (MMVD) is influenced by the method used to estimate left ventricular volume. This study aimed to evaluate the impact of different left ventricular volume estimation methods on quantitative MR assessment, using the modified Simpson’s method of discs (Disc method) as a reference. Echocardiographic data from 167 dogs with MMVD and 19 healthy control dogs were analyzed. Regurgitant volume (RVol), body size-normalized RVol, and regurgitant fraction (RF) were calculated using diameter-based methods (Cube, Gibson, Meyer, and Teichholz) and compared with values obtained using the Disc method. All diameter-based methods showed significant positive correlations with the Disc method. However, Bland–Altman analyses demonstrated wide limits of agreement and systematic bias. Between-method discrepancies increased with advancing disease stage, with diameter-based methods tending to overestimate RVol and RF, particularly in dogs classified as American College of Veterinary Internal Medicine (ACVIM) stages B2 and C/D. Although relative trends in regurgitant indices were consistent across methods, substantial differences were observed in absolute values. These findings indicate that diameter-based methods are not interchangeable with the Disc method for absolute quantification of MR severity in dogs with MMVD, especially in advanced disease stages.

## Simple Summary

Myxomatous mitral valve disease is the most common heart disease in dogs and causes blood to leak backward through the mitral valve during each heartbeat. This backward leakage increases the workload of the heart and can eventually lead to heart failure. In human cardiology, the severity of valve leakage is commonly assessed using quantitative measurements that estimate how much blood flows backward and how much fails to move forward from the heart into the body. In veterinary medicine, similar quantitative approaches are being investigated; however, their interpretation depends greatly on how the size of the left ventricle, the main pumping chamber of the heart, is calculated. Traditionally, several simplified methods have been used to estimate heart size from a single internal diameter, mainly because they require fewer measurements and less complex image analysis. In this study, we compared these conventional simplified methods with a more detailed approach that calculates heart volume using multiple two-dimensional ultrasound images. Although the simplified methods showed similar overall trends, they produced different absolute values, especially in dogs with more advanced heart disease. These findings indicate that caution is needed when simplified quantitative methods are used to assess the severity of heart valve leakage in dogs.

## 1. Introduction

Myxomatous mitral valve disease (MMVD) is the most common acquired cardiac disease in dogs. It is characterized by progressive myxomatous degeneration of the mitral valve, which results in mitral regurgitation (MR) [1]. As MR progresses, chronic volume overload of the left atrium and left ventricle results in cardiac chamber enlargement, increased filling pressures, and ventricular remodeling. These pathophysiological changes are closely associated with the onset of congestive heart failure and overall prognosis. Therefore, accurate assessment of MR severity is of major clinical importance.

The American College of Veterinary Internal Medicine (ACVIM) has proposed consensus guidelines for the diagnosis and management of MMVD, with disease stage being primarily determined based on the presence of cardiac enlargement and clinical signs of heart failure [2]. However, this staging system mainly relies on structural changes and does not necessarily reflect the hemodynamic burden directly imposed by MR. In fact, some dogs exhibit marked MR despite minimal cardiac chamber enlargement, including cases with high regurgitant fraction (RF) values [3]. This indicates that reliance on structural indices alone may lead to underestimation of MR severity in certain dogs.

In human cardiology, regurgitant volume (RVol) and RF are widely used quantitative indices for assessing the hemodynamic severity of MR [4]. These indices can be calculated using several techniques, including volumetric methods based on left ventricular volume measurements and the proximal isovelocity surface area method [5]. Among these approaches, volumetric methods enable comprehensive assessment of total MR volume based on left ventricular end-diastolic volume (LVEDV) and end-systolic volume (LVESV). According to guidelines from the American Society of Echocardiography, an RF ≥ 50% is considered indicative of severe MR in humans [6]. However, the accuracy of RVol and RF derived from volumetric methods is highly dependent on the accuracy of left ventricular volume estimation [7,8,9].

Several simplified methods have been proposed to estimate left ventricular volume using one-dimensional measurements of the left ventricular internal diameter, including the Cube, Gibson, Meyer, and Teichholz methods, which are largely derived from M-mode echocardiography [10,11,12,13]. Although these methods are relatively easy to perform, they rely on geometric assumptions that approximate the left ventricle as a prolate ellipsoid or spherical structure [7].

In contrast, the modified Simpson’s method of discs (Disc method), which is based on two-dimensional echocardiography, allows volumetric assessment of the left ventricle using multiple cross-sectional images [14]. This approach requires fewer geometric assumptions and is considered more suitable for estimating left ventricular volume in dogs with ventricular dilation or asymmetric remodeling. Accordingly, the Disc method is commonly regarded as a reference technique, despite its reliance on adequate image quality and operator expertise [15,16].

As MMVD progresses, the left ventricle undergoes asymmetric dilation and geometric remodeling rather than uniform enlargement [14]. Under these conditions, the accuracy of left ventricular volume estimation methods based on a single diameter remains uncertain. Moreover, differences among volume estimation methods may influence not only absolute volume measurements but also derived quantitative indices such as RVol and RF, potentially complicating interpretation of MR severity and comparisons among studies.

Although the use of RVol and RF for quantitative assessment of MR in dogs with MMVD has been reported, the extent to which different left ventricular volume estimation methods affect these indices relative to the Disc method has not been fully clarified.

Therefore, the aim of this study was to compare RVol and RF calculated using left ventricular volumes estimated by commonly used diameter-based methods (Cube, Gibson, Meyer, and Teichholz) with those obtained using the Disc method as a reference in dogs with MMVD, and to evaluate the agreement and systematic differences among these methods.

## 2. Materials and Methods

### 2.1. Ethics Statement

This retrospective cohort study was approved by the ethics committee of Nippon Veterinary and Life Science University (approval number: R2–5 and 25–07). The animal owners provided written informed consent for participation in this study.

### 2.2. Study Design and Animals

We included dogs diagnosed with MMVD and underwent echocardiographic examination at the Veterinary Medical Center of Nippon Veterinary and Life Science University or the Veterinary Teaching Hospital of Okayama University of Science between 1 April 2023 and 31 July 2024. The diagnosis of MMVD was based on characteristic echocardiographic findings, including thickening and/or prolapse of the mitral valve leaflets with associated mitral regurgitation detected by color Doppler imaging. Comprehensive echocardiographic assessment included two-dimensional echocardiography, color Doppler imaging, pulsed-wave Doppler, and continuous-wave Doppler examinations [17,18].

Dogs were excluded if they had congenital cardiac diseases (e.g., atrial septal defect, ventricular septal defect, and patent ductus arteriosus) or primary structural or myocardial diseases unrelated to MMVD, including mitral valve dysplasia, infective endocarditis, cardiomyopathies, primary pulmonary hypertension, or clinically significant arrhythmias. Secondary cardiac changes attributable to MMVD progression, such as pulmonary hypertension or tricuspid regurgitation, were not considered exclusion criteria. Dogs with aortic outflow showing high-velocity turbulent flow suggestive of subaortic stenosis or other outflow obstruction were also excluded. Cases with insufficient image quality for reliable volumetric measurements were excluded. Diagnostic specificity for MMVD was ensured based on signalment, clinical history, physical examination findings, and echocardiographic morphology. Thoracic radiography was performed in dogs with suspected or confirmed cardiac remodeling or congestive heart failure to assess cardiac enlargement and pulmonary edema in accordance with ACVIM staging criteria. Cardiac biomarkers (e.g., N-terminal pro-B-type natriuretic peptide or cardiac troponin) were not systematically evaluated in all dogs and were therefore not used as inclusion criteria. Healthy control dogs were defined as dogs without an audible heart murmur and without structural or functional abnormalities on echocardiographic examination. Echocardiographic examinations in control dogs were performed as part of pre-anesthetic screening or detailed cardiac evaluation.

### 2.3. Classification

Dogs with MMVD were classified into stages B1, B2, and C/D according to the 2019 consensus guidelines of the ACVIM, which are based on clinical signs, thoracic radiographic findings, echocardiographic evidence of cardiac enlargement, and history of treatment for congestive heart failure [2]. We collected data regarding age, sex, and body weight. Further, we reviewed previous cardiovascular examination results and treatment histories, when available.

### 2.4. Echocardiography Examination

We retrospectively analyzed echocardiographic examinations performed as part of routine clinical assessment. Examinations were conducted at two institutions using the same diagnostic ultrasonographic system (Vivid E95; Vivid E95 Ultra Edition; GE Healthcare, Tokyo, Japan) equipped with phased-array transducers. Acquisition settings were standardized within each institution. Image acquisition was performed by experienced cardiology clinicians at each institution and followed the standard right parasternal and left apical views [19,20]. Three consecutive cardiac cycles were recorded under sinus rhythm during end expiration with simultaneous lead II electrocardiogram monitoring. End expiration was visually identified based on minimal thoracic wall motion. All images were digitally stored and subsequently re-analyzed offline using commercially available software (EchoPAC, version 204; GE Healthcare, Tokyo, Japan). Offline measurements were performed independently by two investigators (S.K. and Y.M.), each responsible for analysis of cases from their respective institutions. All measurements were performed following standardized measurement protocols under cardiology supervision. Both investigators were blinded to ACVIM stage classification. For each parameter, measurements were obtained from three consecutive cardiac cycles and averaged for analysis.

### 2.5. Left Ventricular Volume Measurements

LVEDV and LVESV were calculated using the Disc method, Cube method, Gibson method, Meyer method, and Teichholz method [10,11,12,13,14]. Regarding the disc summation method, LVEDV and LVESV were obtained by manual tracing of the left ventricular endocardial border from the left apical long-axis view at end-diastole and end-systole, in accordance with the American Society of Echocardiography chamber quantification guidelines [21]. End-diastole was defined as the frame at the onset of the QRS complex or the frame in which the left ventricular cavity dimension was maximal. End-systole was defined as the frame immediately following aortic valve closure or the frame in which the left ventricular cavity dimension was minimal. Care was taken to avoid foreshortening by ensuring visualization of the true left ventricular apex and maximal longitudinal axis. Papillary muscles and trabeculations were excluded from the ventricular cavity.

**Figure 1:**
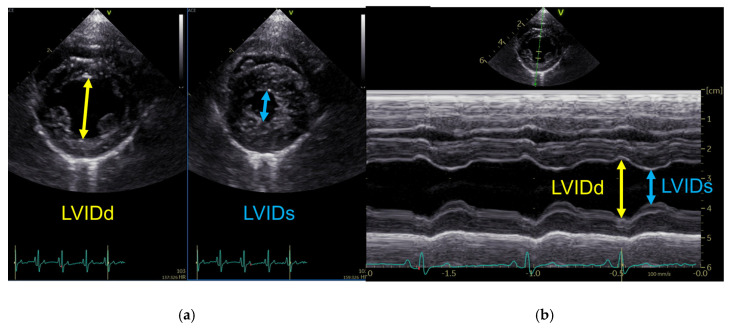
Representative echocardiographic images illustrating left ventricular internal diameter measurements used for diameter-based volume estimation. (**a**) Two-dimensional measurement of LVID from the right parasternal short-axis view at end-diastole (LVIDd) and end-systole (LVIDs). (**b**) M-mode measurement of LVIDd and LVIDs obtained from the right parasternal short-axis view at the level of the chordae tendineae. Yellow arrows indicate LVIDd, and blue arrows indicate LVIDs.

Contrastingly, the Cube, Gibson, Meyer, and Teichholz methods estimate the left ventricular volume from one-dimensional measurements of the left ventricular internal diameter (LVID). LVID in diastole (LVIDd) and systole (LVIDs) were measured from the right parasternal short-axis view at the level of the chordae tendineae, primarily using M-mode recordings. When optimal cursor alignment perpendicular to the interventricular septum and left ventricular free wall could not be achieved, measurements were obtained from two-dimensional images to avoid geometric distortion. As shown in Table 1, left ventricular volumes were calculated using established diameter-based equations. The measurement principles for LVID measurements used for diameter-based volume estimation are shown in Figure 1. All volumetric measurements were performed offline by two investigators (S.K. and Y.M.), each responsible for cases from their respective institutions, and averaged over three consecutive cardiac cycles.

### 2.6. Calculation of Mitral Regurgitant Volume and Regurgitant Fraction

Left ventricular stroke volume (LVSV) was calculated for each method as the difference between left ventricular end-diastolic volume (LVEDV) and end-systolic volume (LVESV). Left ventricular outflow tract stroke volume (LVOT SV) was calculated as the product of the left ventricular outflow tract cross-sectional area and the velocity–time integral (VTI). The LVOT diameter was measured at mid-systole from the right parasternal long-axis view, and the cross-sectional area was calculated assuming a circular shape. The LVOT VTI was obtained using pulsed-wave Doppler from the left apical five-chamber view [4,19,20]. Mitral RVol was calculated for each method as the difference between the method-specific LVSV and LVOT SV. RF was calculated as the ratio of RVol to LVSV and expressed as a percentage [4]. To account for differences in body size, RVol was further normalized to body weight (RVol/kg) and body surface area (RVol/BSA) [15,22]. All Doppler and LVOT measurements were performed offline by the same investigators (S.K. and Y.M.), each responsible for cases from their respective institutions, and averaged over three consecutive cardiac cycles.

### 2.7. Statistical Analysis

Statistical analyses were performed using R (version 2.8.1; The R Foundation for Statistical Computing, Vienna, Austria). Continuous variables are expressed as median and interquartile range, while categorical variables are expressed as number and percentage. Normality of data distribution was assessed using the Shapiro–Wilk test. Since most variables showed a non-normal distribution, non-parametric statistical methods were applied for subsequent analyses.

Bland–Altman analysis was used to evaluate the agreement between the disc summation method and each diameter-based method with respect to RVol, RVol/kg, RVol/BSA, and RF. Mean differences (bias) and 95% limits of agreement (LOA), defined as bias ± 1.96 standard deviations, were calculated. Bland–Altman plots were generated to visually assess the relationship between the measurement magnitude and method-specific differences.

Additionally, Spearman’s rank correlation coefficients were used to evaluate monotonic associations between measurements obtained by the disc summation method and each diameter-based method. Correlation analyses were performed for dogs with ACVIM stage B1–C/D MMVD. Supplementary Tables S5–S7 present a complete correlation matrix.

Comparisons of continuous variables among ACVIM stages were performed using the Kruskal–Wallis test, followed by post hoc pairwise comparisons using the Steel–Dwass test, when appropriate. A *p* value < 0.05 was considered statistically significant.

To assess measurement variability, repeatability was evaluated using intraclass correlation coefficient (ICC) in a subset of dogs with MMVD. A total of 9 dogs were randomly selected, including 3 dogs each from ACVIM stages B1, B2, and C/D, to ensure representation across disease severity. Repeatability was assessed for key variables used in volumetric calculations, including LVEDV, LVESV, LVOT diameter, and LVOT VTI. All measurements for repeatability analysis were performed independently by two observers (S.K. and A.K.) who were blinded to each other’s and their own previous results. ICC values were calculated using a two-way random-effects model for absolute agreement.

## 3. Results

### 3.1. Study Population

During the study period, 195 dogs underwent echocardiographic examination. Nine dogs were excluded because of inadequate image quality (n = 5) or the presence of aortic stenosis with high-velocity turbulent flow (n = 4).

Accordingly, we included 186 dogs (19 healthy controls and 167 dogs diagnosed with MMVD). Among the 167 dogs with MMVD, 83, 57, and 27 dogs were classified as stage B1, stage B2, and stage C/D, respectively.

The study population (including both healthy control dogs and dogs with MMVD) consisted predominantly of small-breed dogs, including Chihuahuas, Toy Poodles, Miniature Dachshunds, Pomeranians, Shih Tzus, and Cavalier King Charles Spaniels. A small number of sighthound-type breeds (e.g., Borzoi and Italian Greyhound) were also included. The distribution of dog breeds is presented in Supplementary Table S1.

Echocardiographic findings in dogs with MMVD were consistent with typical features of the disease.

Age and body weight showed significant among-group differences, with dogs in the B2 and C/D groups being older and having lower body weight compared with control dogs. Echocardiographic variables, including fractional shortening, left atrial-to-aortic ratio, and body-weight-normalized left ventricular internal diameter in diastole, showed a progressive increase with advancing ACVIM stage (Table 2). Contrastingly, left ventricular internal diameters only showed partial among-group differences and did not differ significantly between the control and B1 groups. LVID measurements were obtained using M-mode in 56 dogs and two-dimensional imaging in 130 dogs, with two-dimensional measurements used when optimal M-mode alignment could not be achieved due to variations in cardiac axis and imaging conditions.

### 3.2. Distribution of Mitral Regurgitation Parameters Calculated Using the Modified Simpson’s Method of Discs

**Figure 2:**
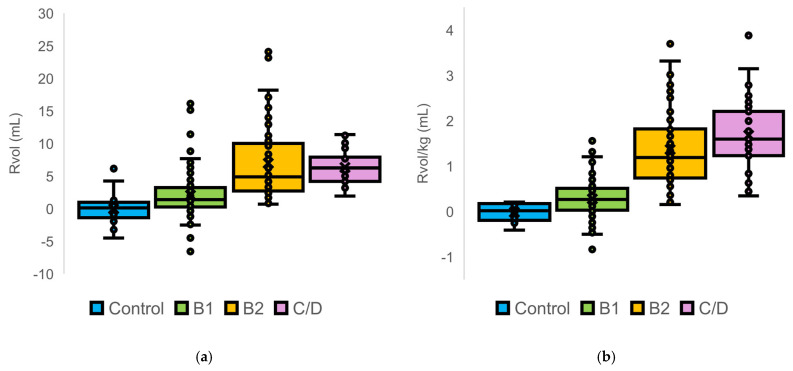

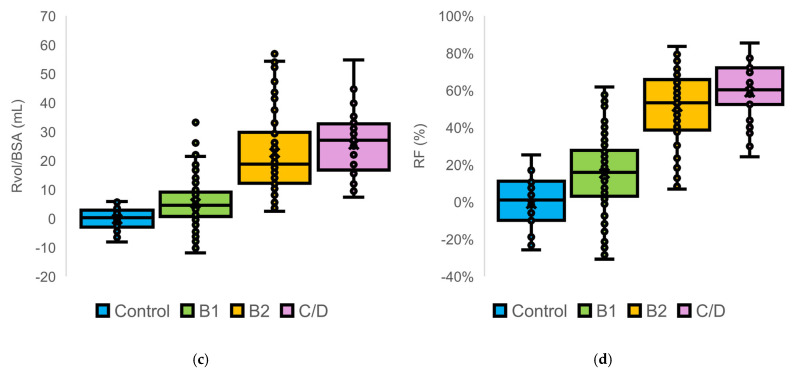
Distribution of quantitative mitral regurgitation parameters calculated using the modified Simpson’s method of discs in dogs stratified by American College of Veterinary Internal Medicine stage: (**a**) regurgitant volume; (**b**) body weight-normalized regurgitant volume; (**c**) body surface area-normalized regurgitant volume; and (**d**) regurgitant fraction. Boxes represent median values and interquartile ranges, whiskers indicate 1.5 × the interquartile range, and dots represent individual dogs.

We performed among-stage comparisons of the distributions of MR parameters calculated using the Disc method (Figure 2).

Median values of RVol, RVol/kg, RVol/BSA, and RF increased stepwise with advancing ACVIM stage, which was accompanied by a progressive widening of data dispersion. This trend was particularly pronounced in the B2 and C/D groups. In control dogs, RF values were centered around zero and ranged from −26% to 25%. Negative RF values were observed in control and some B1 dogs, whereas all RF values in the B2 and C/D groups were positive.

### 3.3. Agreement Between the Modified Simpson’s Method of Discs and Diameter-Based Methods

#### 3.3.1. Regurgitant Volume

**Figure 3:**
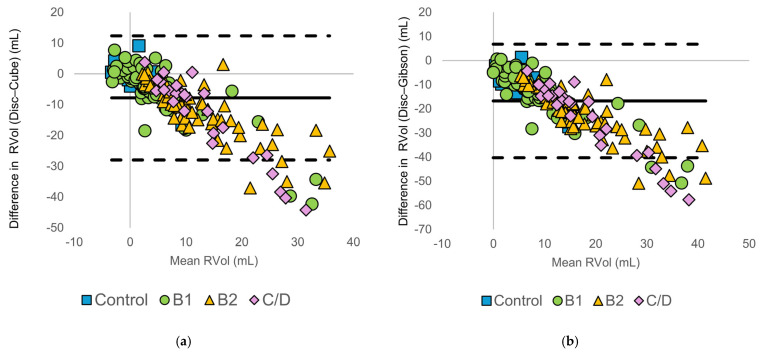

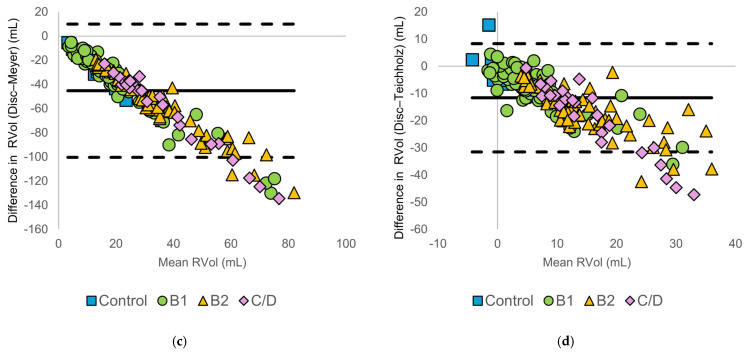
Bland–Altman plots comparing regurgitant volume (RVol) calculated using the modified Simpson’s method of discs (Disc method) and diameter-based echocardiographic methods: (**a**) Disc vs. Cube, (**b**) Disc vs. Gibson, (**c**) Disc vs. Meyer, and (**d**) Disc vs. Teichholz. The solid horizontal line represents the mean bias, and the dashed horizontal lines indicate the 95% limits of agreement. Symbols denote American College of Veterinary Internal Medicine (ACVIM) stages (control, B1, B2, and C/D).

Bland–Altman analysis was used to evaluate the agreement between the disc summation method and each diameter-based method (Figure 3).

For all diameter-based methods, differences from the disc summation method increased with advancing ACVIM stage. Specifically, diameter-based methods frequently yielded higher RVol values than the disc summation method in the B2 and C/D groups. Bland–Altman plots demonstrated a progressive increase in negative bias and widening limits of agreement with increasing mean RVol, indicating that agreement between methods deteriorated as regurgitant volume increased. A summary of the Bland–Altman statistics, including bias and LOA, is presented in Supplementary Table S2.

#### 3.3.2. Body Size-Normalized Regurgitant Volume

**Figure 4:**
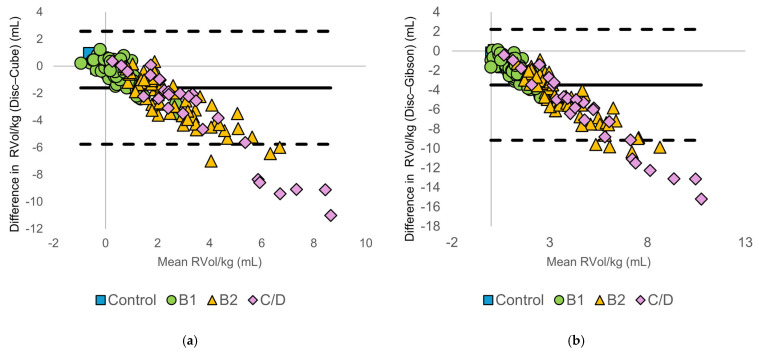

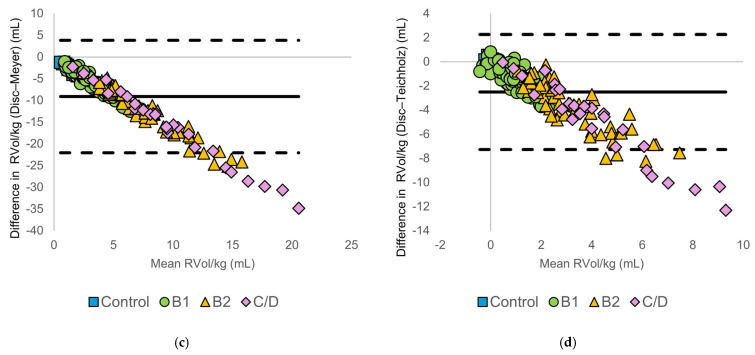
Bland–Altman plots comparing body weight-normalized regurgitant volume (RVol/kg) calculated using the modified Simpson’s method of discs (Disc method) and diameter-based echocardiographic methods: (**a**) Disc vs. Cube, (**b**) Disc vs. Gibson, (**c**) Disc vs. Meyer, and (**d**) Disc vs. Teichholz. The solid horizontal line represents the mean bias, and the dashed horizontal lines indicate the 95% limits of agreement. Symbols denote American College of Veterinary Internal Medicine (ACVIM) stages (control, B1, B2, and C/D).

**Figure 5:**
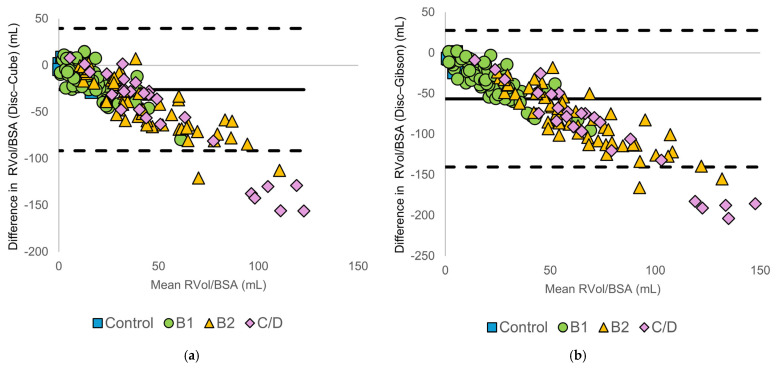

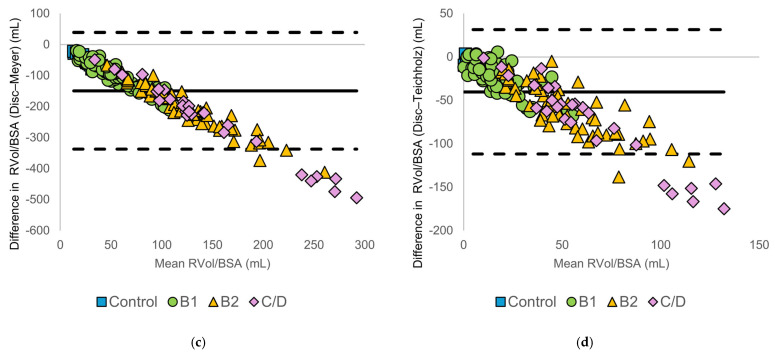
Bland–Altman plots comparing body surface area-normalized regurgitant volume (RVol/BSA) calculated using the modified Simpson’s method of discs (Disc method) and diameter-based echocardiographic methods: (**a**) Disc vs. Cube, (**b**) Disc vs. Gibson, (**c**) Disc vs. Meyer, and (**d**) Disc vs. Teichholz. The solid horizontal line represents the mean bias, and the dashed horizontal lines indicate the 95% limits of agreement. Symbols denote American College of Veterinary Internal Medicine (ACVIM) stages (control, B1, B2, and C/D).

Normalization to body weight or body surface area did not reduce method-specific differences. Bland–Altman plots (Figure 4 and Figure 5) demonstrated increasing negative bias and widening limits of agreement with increasing RVol/kg and RVol/BSA. These findings indicate that discrepancies between methods increased with higher regurgitant volumes, regardless of body size. Discrepancies between the disc summation method and diameter-based methods remain evident, particularly in the C/D group. A summary of the Bland–Altman statistics, including bias and limits of agreement, is presented in Supplementary Tables S3 and S4.

#### 3.3.3. Regurgitant Fraction

**Figure 6:**
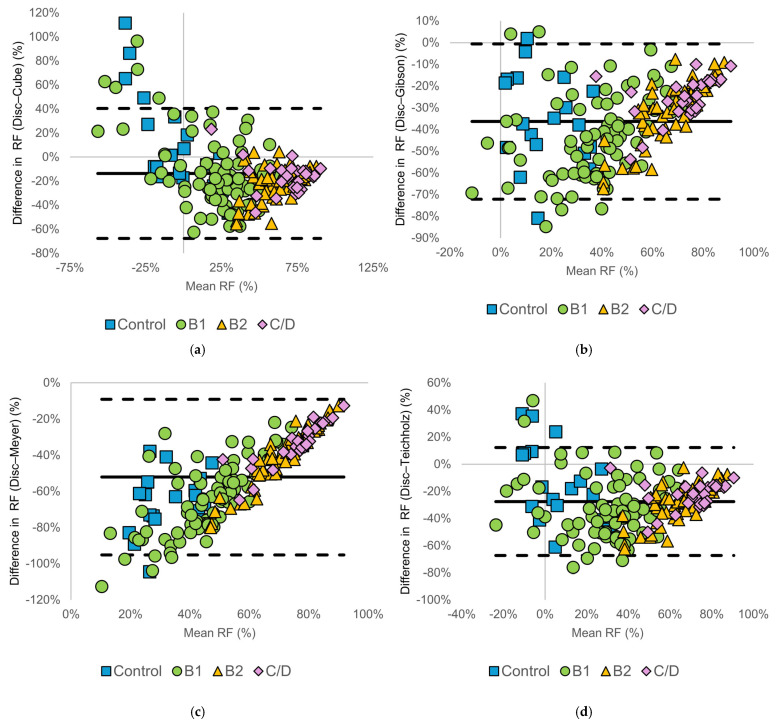
Bland–Altman plots comparing regurgitant fraction (RF) calculated using the modified Simpson’s method of discs (Disc method) and diameter-based echocardiographic methods: (**a**) Disc vs. Cube, (**b**) Disc vs. Gibson, (**c**) Disc vs. Meyer, and (**d**) Disc vs. Teichholz. The solid horizontal line represents the mean bias, and the dashed horizontal lines indicate the 95% limits of agreement. Symbols denote American College of Veterinary Internal Medicine (ACVIM) stages (control, B1, B2, and C/D).

Figure 6 shows the Bland–Altman analysis for RF. Among dogs with ACVIM stage B2 and C/D disease, differences between the disc summation method and diameter-based methods were relatively less dispersed compared with RVol-based indices. However, diameter-based methods consistently yielded higher RF values than the disc summation method. Notably, the direction and magnitude of differences varied among methods. In the Cube and Teichholz methods, differences were distributed around zero with both positive and negative values, whereas in the Gibson and Meyer methods, differences were predominantly negative. This reflects method-specific differences in left ventricular volume estimation, with some diameter-based methods producing greater overestimation of left ventricular volumes, which in turn affects both stroke volume and RF calculations. In control dogs, RF values were highly variable, with between-method differences showing wide distributions in both positive and negative directions and no clear association. Summary statistics of the Bland–Altman analyses, including bias and LOA for each method and ACVIM stage, are presented in Table 3.

### 3.4. Correlation

For RVol, there were significant positive correlations between the disc summation method and all diameter-based methods (r = 0.63–0.71, all *p* < 0.05). Further, there were extremely strong correlations among diameter-based methods themselves (r = 0.97–1.0, all *p* < 0.05).

Similar results were obtained for RVol/kg (r = 0.67–0.76 for disc summation vs. diameter-based methods; all *p* < 0.05), with correlations among diameter-based methods remaining high (r = 0.94–1.0).

RF values showed higher correlations between the disc summation method and diameter-based methods than those observed for RVol-based indices (r = 0.82–0.84, all *p* < 0.05). Correlations among diameter-based methods remained very strong (r = 0.97–1.0). Supplementary Tables S5–S7 present the correlation matrices.

### 3.5. Measurement Repeatability

Repeatability analysis demonstrated good to excellent agreement for all measured variables. ICC values were high for LVEDV, LVESV, LVOT diameter, and LVOT VTI, as shown in Supplementary Table S8.

## 4. Discussion

We evaluated the impact of different left ventricular volume estimation methods on quantitative assessment of MR in dogs with MMVD, using the modified Simpson’s method of discs as the reference standard [14]. RVol, RVol/BSA, RVol/kg, and RF calculated using diameter-based methods showed significant positive correlations with values obtained using the disc summation method. However, Bland–Altman analyses demonstrated wide LOA and method-specific systematic bias across all indices. Notably, between-method discrepancies increased with advancing ACVIM stage, with diameter-based methods tending to overestimate the RVol and RF compared with the disc summation method in dogs with more advanced disease (ACVIM stages B2 and C/D). These findings indicate that, despite similar directional changes with disease progression, diameter-based methods are not interchangeable with the disc summation method for absolute quantification of MR severity in dogs with MMVD.

The observed between-method discrepancies can be primarily attributed to differences in the geometric assumptions underlying left ventricular volume estimation [8]. The Cube, Gibson, Meyer, and Teichholz methods estimate ventricular volume from a single linear dimension and assume a simplified ventricular geometry, typically approximating the left ventricle as a sphere, cylinder, or rotational ellipsoid [10,11,12,13]. Accordingly, these methods heavily rely on left ventricular internal diameter measurements obtained from the right parasternal short-axis view.

Contrastingly, the disc summation method calculates the left ventricular volume by dividing the ventricle into multiple discs and summing their volumes based on endocardial tracings obtained from the left apical long-axis view [14]. This approach requires fewer geometric assumptions and is better suited to capture regional variations in ventricular shape, asymmetrical remodeling, and localized chamber dilation.

In dogs with MMVD, progressive volume overload induces left ventricular dilation accompanied by increasing sphericity and geometric distortion. These disease-related changes involve asymmetric and non-uniform ventricular remodeling rather than simple proportional enlargement of the left ventricle [23,24]. Such deviations from idealized ventricular geometry are not adequately represented by one-dimensional diameter-based models, especially in the advanced disease stages, which results in progressive divergence from the disc summation method as well as leads to the systematic bias and widening LOA observed in our study. The high repeatability observed for key measurement variables suggests that the discrepancies between methods are unlikely to be solely attributable to measurement variability, but rather reflect inherent differences in volume estimation approaches. This supports the interpretation that method-dependent bias observed in Bland–Altman analyses represents true systematic differences rather than random measurement error.

The observed increase in between-method discrepancies with advances in disease stage has important clinical implications. In our study, dogs in ACVIM stages B2 and C/D groups showed progressively larger between-method differences, with diameter-based approaches tending to overestimate both RVol and RF compared with the disc summation method. Because these stages correspond to clinically relevant thresholds for therapeutic decision-making, such discrepancies may influence the interpretation of MR severity in advanced disease. Previous studies have shown that enlargement and geometric remodeling of the left ventricle increase the error associated with one-dimensional diameter-based volume estimation in dogs with cardiac disease [8]. Our findings suggest that these geometric limitations may also contribute to discrepancies in MR quantification when diameter-based methods are applied in dogs with advanced MMVD.

From a practical standpoint, diameter-based methods remain widely used in routine veterinary echocardiography because they require fewer measurements and can be applied rapidly during standard examinations. In clinical environments where time, equipment, or operator experience may be limited, these simplified approaches may provide a practical option for monitoring relative changes in MR severity during serial examinations, particularly when the same method is applied consistently. In such situations, M-mode-derived or other diameter-based indices may represent a practical approach for longitudinal follow-up in general clinical practice. Accordingly, diameter-based indices may be acceptable for longitudinal follow-up within the same patient, whereas volumetric methods may be preferable when more accurate absolute quantification of MR severity is required.

At present, there is no universally established threshold for clinically acceptable measurement error for volumetric MR quantification in dogs. Nevertheless, the magnitude of bias and LOA observed in the present study suggests that discrepancies between methods may exceed ranges that would be considered clinically negligible when threshold-based indices are used to guide therapeutic decisions. Therefore, caution may be warranted when different volumetric methods are used interchangeably in clinical decision-making, particularly when absolute MR severity thresholds are considered.

Future technological developments, including automated image analysis and artificial intelligence-assisted echocardiographic tools, may help improve the reproducibility and standardization of volumetric measurements. Such approaches may reduce operator-dependent variability in endocardial border tracing and volumetric calculations. However, the performance of these technologies will still depend on adequate image acquisition and image quality, which remain operator-dependent in veterinary echocardiography.

In our study, RF showed stronger correlations between the disc summation method and diameter-based methods than RVol-based indices. This can be attributed to the fact that RF is calculated as the ratio of RVol to LVSV, with both the numerator and denominator being derived from the same volume estimation method. Accordingly, systematic errors introduced by geometric assumptions in left ventricular volume estimation may partially cancel each other out, which results in improved rank-order agreement between methods.

Despite this relatively strong correlation, Bland–Altman analysis revealed wide LOA and persistent systematic bias for RF, especially at higher values. This indicates that, although RF values calculated using different methods may change in a similar direction with disease progression, their absolute values are not interchangeable. This discrepancy arises because RF is derived from both LVSV and LVOT SV, and method-specific differences in left ventricular volume estimation can propagate nonlinearly into RF calculations. Method-specific overestimation or underestimation of left ventricular volumes can propagate into RF calculations, resulting in clinically meaningful discrepancies even when correlation coefficients appear high.

Furthermore, RF values in control dogs and some dogs in stage B1 were distributed around zero, with a bidirectional range that included negative values. This does not indicate negative regurgitation, a physiologic impossibility, but rather reflects the limitations of the volumetric subtraction method in the presence of measurement error and inherent methodological assumptions in volume estimation. This can be explained by the calculation of RF in this study. RF was calculated as the difference between two independently measured stroke volumes (left ventricular stroke volume and LVOT stroke volume) divided by left ventricular stroke volume. When the true regurgitant volume is minimal or absent, small discrepancies in endocardial tracing, diameter measurement, or Doppler-derived LVOT VTI can be proportionally amplified, resulting in calculated values slightly above or below zero. In contrast, as disease severity increased and true regurgitant volume became substantial (B2 and C/D stages), RF values shifted consistently into the positive range, and negative values were no longer observed. This stage-dependent distribution pattern supports the interpretation that negative RF values in control and stage B1 dogs primarily reflect measurement variability inherent to subtraction-based volumetric calculations rather than physiological phenomena. Similar observations have been reported in previous studies, in which negative or unexpectedly variable RF values were described in dogs with stage B1 MMVD or in dogs without detectable MR when volumetric subtraction methods were applied [15]. Accordingly, RF should be interpreted with caution when true regurgitant volume is expected to be very small, as negative RF values reflect measurement error rather than a physiological phenomenon.

RVol/kg or RVol/BSA did not show improved between-method agreement. Although body size normalization is commonly used to facilitate comparison across individuals with different body sizes, such scaling primarily adjusts the magnitude of measured values and does not address the fundamental source of error inherent in left ventricular volume estimation.

The observed between-method discrepancies were mainly driven by differences in geometric assumptions and volume calculation algorithms rather than inter-individual variability in body size. Consequently, normalization of RVol did not correct method-specific systematic bias; moreover, widening LOA persisted even after adjustment for body weight or body surface area, particularly in dogs with advanced MMVD.

These findings indicate that body size-normalized RVol indices do not confer interchangeability between volume estimation methods; accordingly, this normalization does not compensate for inaccuracies in left ventricular volume measurement. Taken together, when comparing quantitative MR parameters across studies or clinical settings, it is important to carefully consider the underlying volume estimation method, regardless of whether body size normalization is applied.

This study has several limitations. First, this was a retrospective observational study, and image acquisition conditions were not fully standardized. Accordingly, we could not strictly control some factors that may influence left ventricular volume estimation and flow measurements, including heart rate, loading conditions, and blood pressure, which may have contributed to measurement variability. In addition, intraobserver and interobserver variability were not formally evaluated in this study. Variability related to image acquisition, endocardial border tracing, and Doppler measurements may therefore have contributed to measurement differences.

Second, although the disc summation method was used as the reference method, it does not represent a true gold standard for quantifying MR. This method relies on two-dimensional echocardiographic images and may be affected by image quality, endocardial border delineation, foreshortening, and geometric distortion in severely remodeled ventricles. Therefore, some of the observed between-method discrepancies may reflect limitations of the disc summation method itself rather than inaccuracies of diameter-based methods. Three-dimensional imaging modalities, such as three-dimensional echocardiography, cardiac magnetic resonance imaging, and cardiac computed tomography, are less dependent on geometric assumptions and may provide more direct and potentially more accurate assessments of left ventricular volumes [25,26]. By reducing reliance on single-plane two-dimensional imaging, these techniques may mitigate errors related to foreshortening and ventricular geometric modeling. However, these modalities are not routinely available in all clinical settings and often require specialized equipment and, in the case of magnetic resonance imaging or computed tomography, general anesthesia in dogs. Future prospective studies incorporating these three-dimensional techniques are warranted to further validate quantitative MR assessment methods in dogs with MMVD. Another limitation of this study is that left ventricular measurements were obtained from different echocardiographic views depending on the method used. Diameter-based methods relied on measurements from the right parasternal short-axis view, whereas the disc summation method, which served as the reference standard in this study, was based on tracings from the left apical long-axis view. This difference in imaging planes may introduce an additional source of variability when comparing methods, and therefore part of the observed discrepancies may reflect differences in image acquisition rather than volume estimation algorithms alone.

Third, the relatively small number of dogs with advanced disease (ACVIM stage C/D) represents an additional limitation of this study. Because only 27 dogs were included in this group, the statistical precision of estimates for advanced stages may be limited. In addition, dogs in ACVIM stages C and D were analyzed together because the number of dogs in each subgroup was insufficient to allow meaningful statistical comparison between these stages.

Finally, cases were obtained from referral institutions, which may introduce referral bias. Dogs referred to specialty centers may not fully represent the broader population of dogs with MMVD encountered in general veterinary practice, and therefore caution may be required when generalizing the present findings to the wider clinical population.

Taken together, these findings suggest that volumetric methods based on the disc summation method may provide more reliable estimates of MR severity in dogs with advanced MMVD, whereas diameter-based methods may be more appropriate for longitudinal follow-up when applied consistently within the same patient.

## 5. Conclusions

In dogs with MMVD, quantitative MR indices derived from different left ventricular volume estimation methods are not interchangeable. Although diameter-based methods demonstrated significant correlations with the disc summation method and reflected directional changes in disease progression, bias and LOA worsened with advancing disease severity. Specifically, as RVol increased, the differences between measurement methods became larger across all evaluated methods. In addition, all one-dimensional diameter-based methods systematically overestimated RVol compared with the disc summation method. These discrepancies were not mitigated by normalization to body weight or body surface area.

These findings indicate that quantitative MR parameters calculated using one-dimensional diameter-based methods should be interpreted with increasing caution as disease severity advances. In particular, in dogs with more advanced disease (ACVIM stages B2 and C/D), one-dimensional diameter-based methods may substantially overestimate RVol and should therefore be avoided when accurate absolute quantification is required for clinical decision-making. Although such methods may be useful for longitudinal assessment or relative comparisons when applied consistently within the same patient, the underlying assumptions and limitations of each volume estimation method must be carefully considered. Our findings highlight the importance of method selection in the quantitative evaluation of MR severity in dogs with MMVD.

## Figures and Tables

**Table 1:** Equations used for diameter-based left ventricular volume estimation.

Method	Equation
Cube method	$Left\;ventricular\;volume=D^{3}$
Gibson method	$Left\;ventricular\;volume=0.16\pi D^{3}+0.98\pi D^{2}$
Meyer method	$Left\;ventricular\;volume=0.62D^{3}+14.6D-19.1$
Teichholz method	$Left\;ventricular\;volume=7.0D^{3}/(2.4+D)$

D represents the left ventricular internal diameter (LVIDd or LVIDs) measured at the level of the chordae tendineae from the right parasternal short-axis view at end-diastole or end-systole, expressed in centimeters.

**Table 2:** Baseline characteristics and echocardiographic variables in dogs with myxomatous mitral valvular disease stratified by American College of Veterinary Internal Medicine stage.

Variables	Control (n = 19)	B1 (n = 83)	B2 (n = 57)	C/D (n = 27)
Age (years)	6.3 (3.2, 11.1)	11.3 (8.7, 12.6) *	11.9 (10.1, 13.4) *	12.5 (12.0, 13.6) *#
Sex (Female/Male)	6/13	42/41	28/29	21/6
Body weight (kg)	7.8 (4.7, 12.8)	5.5 (4.0, 7.7) *	4.5 (3.1, 6.3) *#	3.2 (2.9, 6.2) *#
FS (%)	32.9 (28.6, 39.6)	42.0 (35.1, 51.0) *	51.5 (46.4, 59.6) *#	55.7 (51.5, 61.4) *#
LA/Ao	1.1 (1.1, 1.2)	1.3 (1.1, 1.5)	1.9 (1.7, 2.2) *#	2.1 (1.9, 2.5) *#
LVIDDN	1.3 (1.3, 1.5)	1.5 (1.3, 1.6) *	1.8 (1.7, 2.0) *#	1.9 (1.6, 2.4) *#
LVIDd (mm)	25.0 (21.0, 30.2)	24.1 (21.8, 28.7)	30.1 (25.7, 33.6) #	25.9 (24.8, 33.6) #
LVIDs (mm)	17.0 (13.5, 19.6)	14.4 (11.0, 17.1)	14.8 (10.7, 16.9)	12.4 (9.6, 15.5) *

* *p* < 0.05 vs. Control group. # *p* < 0.05 vs. B1 group. FS: fractional shortening; LA/Ao: left atrial-to-aortic ratio; LVIDd: left ventricular internal diameter in diastole; LVIDDN: body-weight-normalized left ventricular internal diameter in diastole; LVIDs: left ventricular internal diameter in systole.

**Table 3:** Summary of Bland–Altman analyses for regurgitant fraction comparing diameter-based methods with the modified Simpson’s method of discs across American College of Veterinary Internal Medicine stages.

Comparison	ACVIM Stage (n)	Bias (%)	Lower LOA (%)	Upper LOA (%)
Disc–Cube	All dogs (n = 186)	−13.6	−67.7	40.4
	Control (n = 19)	12.3	−66.0	90.8
	B1 (n = 83)	−12.9	−73.0	47.2
	B2 (n = 57)	−22.0	−49.7	5.8
	C/D (n = 27)	−16.7	−42.4	9.0
Disc–Gibson	All dogs (n = 186)	−36.3	−72.1	−0.55
	Control (n = 19)	−35.2	−75.4	5.0
	B1 (n = 83)	−43.3	−81.2	19.3
	B2 (n = 57)	−31.4	−59.6	−3.3
	C/D (n = 27)	−25.8	−45.7	−6.0
Disc–Meyer	All dogs (n = 186)	−52.1	−95.1	−9.1
	Control (n = 19)	−65.8	−97.3	−34.3
	B1 (n = 83)	−63.3	−103.0	−23.7
	B2 (n = 57)	−40.3	−72.3	−8.2
	C/D (n = 27)	−33.6	−57.0	−10.2
Disc–Teichholz	All dogs (n = 186)	−27.5	−67.2	12.3
	Control (n = 19)	−14.3	−68.6	40.0
	B1 (n = 83)	−31.9	−76.1	12.3
	B2 (n = 57)	−27.8	−55.1	−0.55
	C/D (n = 27)	−22.7	−43.0	−2.4

Differences were calculated as Disc-diameter-based method. ACVIM: American College of Veterinary Internal Medicine; Disc: modified Simpson’s method of discs; LOA: limits of agreement.

## Data Availability

The datasets used or analyzed in the current study are available from the corresponding author upon reasonable request.

## Supplementary Materials

The following supporting information can be downloaded at: https://www.mdpi.com/article/10.3390/ani16081249/s1, Table S1: Breed distribution of the 186 dogs included in this study. Table S2: Summary of Bland–Altman analyses for RVol comparing diameter-based methods with the modified Simpson’s method of discs across ACVIM stages. Table S3: Summary of Bland–Altman analyses for RVol/kg comparing diameter-based methods with Disc method across ACVIM stages. Table S4: Summary of Bland–Altman analyses for RVol/BSA comparing diameter-based methods with Disc method across ACVIM stages. Table S5: Spearman’s rank correlation coefficients (r) for RVol calculated using the Disc, Cube, Gibson, Meyer, and Teichholz methods in dogs with ACVIM stage B1–C/D MMVD. Table S6: Spearman’s rank correlation coefficients (r) for RVol/kg calculated using the Disc, Cube, Gibson, Meyer, and Teichholz methods in dogs with ACVIM stage B1–C/D MMVD. Table S7: Spearman’s rank correlation coefficients (r) for RF calculated using the Disc, Cube, Gibson, Meyer, and Teichholz methods in dogs with ACVIM stage B1–C/D MMVD. Table S8: ICC values for measurement repeatability of key variables used in volumetric regurgitant volume calculation.
