# Supplementary material for: Comparison and Agreement of Echocardiographic Volumetric Methods for Quantifying Mitral Regurgitation in Dogs with Myxomatous Mitral Valve Disease
Source: Animals (Basel). 2026 Apr 18;16(8):1249. doi: 10.3390/ani16081249 (PMC13113801; doi:10.3390/ani16081249)
Supplement: Supplementary file 1 [file animals-16-01249-s001.zip › animals-4221194-supplementary.pdf]

Supplementary Table S1. Breed distribution of the 186 dogs included in this study.

Breed	Number of dogs
Chihuahua	32
Toy poodle	30
Mixed breed dog	22
Pomeranian	18
Cavalier King Charles Spaniel	18
Miniature dachshund	17
Maltese	8
Miniature Schnauzer	8
Shih tzu	8
Yorkshire terrier	3
Shiba	3
French bulldog	3
Papillon	2
Italian greyhound	2
Boston terrier	2
Jack Russell terrier	1
American cocker spaniel	1
Bichon frise	1
Beagle	1
Welsh corgi	1
Golden retriever	1
Australian shepherd	1
Borzoi	1
Doberman	1
Shikoku	1

Supplementary Table S2. Summary of Bland–Altman analyses for RVol comparing diameter-based methods with the modified Simpson's method of discs across ACVIM stages.

Comparison	ACVIM stage (n)	Bias (mL)	Lower LOA (mL)	Upper LOA (mL)
Disc - Cube	All dogs (n = 186)	-7.8	-28.0	12.4
	Control (n = 19)	-0.23	-10.2	9.7
	B1 (n = 83)	-4.6	-21.1	11.9
	B2 (n = 57)	-12.3	-30.1	5.5
	C/D (n = 27)	-13.3	-39.2	12.5
Disc - Gibson	All dogs (n = 186)	-16.7	-40.3	6.8
	Control (n = 19)	-7.3	-19.9	5.2
	B1 (n = 83)	-12.5	-31.1	6.0
	B2 (n = 57)	-22.6	-43.4	-1.7
	C/D (n = 27)	-23.8	-52.5	-4.9
Disc - Meyer	All dogs (n = 186)	-45.2	-100.4	10.0
	Control (n = 19)	-27.5	-60.8	5.8
	B1 (n = 83)	-35.4	-83.1	12.3
	B2 (n = 57)	-58.5	-108.9	-8.1
	C/D (n = 27)	-59.9	-120.0	0.16
Disc - Teichholz	All dogs (n = 186)	-11.6	-31.5	8.4
	Control (n = 19)	-2.4	-15.3	10.4
	B1 (n = 83)	-7.9	-21.9	6.1
	B2 (n = 57)	-16.8	-34.1	-0.50
	C/D (n = 27)	-18.3	-43.0	6.3

ACVIM, American College of Veterinary Internal Medicine; Disc, modified Simpson's method of discs; LOA, limits of agreement; RVol, regurgitant volume

Supplementary Table S3. Summary of Bland–Altman analyses for RVol/kg comparing diameter-based methods with Disc method across ACVIM stages.

Comparison	ACVIM stage (n)	Bias (mL)	Lower LOA (mL)	Upper LOA (mL)
Disc - Cube	All dogs (n = 186)	-1.6	-5.8	2.6
	Control (n = 19)	0.04	-1.0	1.1
	B1 (n = 83)	-0.61	-2.3	1.1
	B2 (n = 57)	-2.6	-6.0	0.84
	C/D (n = 27)	-3.6	-10.1	2.9
Disc - Gibson	All dogs (n = 186)	-3.5	-9.2	2.2
	Control (n = 19)	-0.89	-2.3	0.49
	B1 (n = 83)	-2.1	-4.4	0.31
	B2 (n = 57)	-5.0	-9.8	-0.19
	C/D (n = 27)	-6.5	-14.3	1.3
Disc - Meyer	All dogs (n = 186)	-9.1	-22.1	3.8
	Control (n = 19)	-3.0	-6.1	0.06
	B1 (n = 83)	-5.8	-11.2	-0.32
	B2 (n = 57)	-12.7	-23.6	-1.9
	C/D (n = 27)	-16.1	-33.0	0.76
Disc - Teichholz	All dogs (n = 186)	-2.5	-7.3	2.3
	Control (n = 19)	-0.37	-1.5	0.80
	B1 (n = 83)	-1.3	-3.3	0.72
	B2 (n = 57)	-3.7	-7.8	0.29
	C/D (n = 27)	-5.0	-11.6	-1.5

ACVIM, American College of Veterinary Internal Medicine; Disc, modified Simpson's method of discs; LOA, limits of agreement; RVol/kg, body weight–normalized regurgitant volume

Supplementary Table S4. Summary of Bland–Altman analyses for RVol/BSA comparing diameter-based methods with Disc method across ACVIM stages.

Comparison	ACVIM stage (n)	Bias (mL)	Lower LOA (mL)	Upper LOA (mL)
Disc - Cube	All dogs (n = 186)	-26.0	-91.5	39.5
	Control (n = 19)	0.19	-19.8	20.1
	B1 (n = 83)	-11.4	-44.2	21.4
	B2 (n = 57)	-42.3	-97.1	12.6
	C/D (n = 27)	-54.8	-154.4	44.7
Disc - Gibson	All dogs (n = 186)	-56.5	-140.5	27.5
	Control (n = 19)	-17.0	-42.3	8.2
	B1 (n = 83)	-36.1	-76.2	4.0
	B2 (n = 57)	-79.9	-148.3	-11.4
	C/D (n = 27)	-97.8	-212.8	17.2
Disc - Meyer	All dogs (n = 186)	-149.2	-337.5	39.2
	Control (n = 19)	-58.7	-109.7	-7.8
	B1 (n = 83)	-101.1	-192.4	-9.9
	B2 (n = 57)	-204.6	-357.2	-52.1
	C/D (n = 27)	-243.5	-487.2	0.18
Disc - Teichholz	All dogs (n = 186)	-40.2	-111.9	31.4
	Control (n = 19)	-6.8	-29.8	16.2
	B1 (n = 83)	-22.9	-57.7	11.8
	B2 (n = 57)	-59.9	-118.7	-1.1
	C/D (n = 27)	-75.5	-173.0	22.0

ACVIM, American College of Veterinary Internal Medicine; Disc, modified Simpson’s method of discs; LOA, limits of agreement; RVol/BSA, body surface area-normalized regurgitant volume

Supplementary Table S5. Spearman's rank correlation coefficients (r) for RVol calculated using the Disc, Cube, Gibson, Meyer, and Teichholz methods in dogs with ACVIM stage B1–C/D MMVD.

RVol	Disc	Cube	Gibson	Meyer	Teichholz
**Disc**	-	0.73 ($p < 0.05$)	0.71 ($p < 0.05$)	0.65 ($p < 0.05$)	0.72 ($p < 0.05$)
**Cube**		-	1.0 ($p < 0.05$)	0.97 ($p < 0.05$)	0.99 ($p < 0.05$)
**Gibson**			-	0.98 ($p < 0.05$)	1.0 ($p < 0.05$)
**Meyer**				-	0.97 ($p < 0.05$)
**Teichholz**					-

ACVIM, American College of Veterinary Internal Medicine; Disc, modified Simpson's method of discs; MMVD, myxomatous mitral valvular disease; RVol, regurgitant volume

Supplementary Table S6. Spearman's rank correlation coefficients (r) for RVol/kg calculated using the Disc, Cube, Gibson, Meyer, and Teichholz methods in dogs with ACVIM stage B1–C/D MMVD.

RVol/kg	Disc	Cube	Gibson	Meyer	Teichholz
**Disc**	-	0.79 ($p < 0.05$)	0.78 ($p < 0.05$)	0.76 ($p < 0.05$)	0.79 ($p < 0.05$)
**Cube**		-	0.98 ($p < 0.05$)	0.96 ($p < 0.05$)	0.98 ($p < 0.05$)
**Gibson**			-	0.99 ($p < 0.05$)	1.0 ($p < 0.05$)
**Meyer**				-	0.99 ($p < 0.05$)
**Teichholz**					-

ACVIM, American College of Veterinary Internal Medicine; Disc, modified Simpson's method of discs; MMVD, myxomatous mitral valvular disease; RVol/kg, body weight–normalized regurgitant volume

Supplementary Table S7. Spearman's rank correlation coefficients (r) for RF calculated using the Disc, Cube, Gibson, Meyer, and Teichholz methods in dogs with ACVIM stage B1–C/D MMVD.

RF	Disc	Cube	Gibson	Meyer	Teichholz
**Disc**	-	0.82 ($p < 0.05$)	0.83 ($p < 0.05$)	0.82 ($p < 0.05$)	0.82 ($p < 0.05$)
**Cube**		-	0.98 ($p < 0.05$)	0.97 ($p < 0.05$)	0.98 ($p < 0.05$)
**Gibson**			-	1.0 ($p < 0.05$)	1.0 ($p < 0.05$)
**Meyer**				-	1.0 ($p < 0.05$)
**Teichholz**					-

ACVIM, American College of Veterinary Internal Medicine; Disc, modified Simpson's method of discs; MMVD, myxomatous mitral valvular disease; RF, regurgitant fraction

Supplementary Table S8. ICC values for measurement repeatability of key variables used in volumetric regurgitant volume calculation.

Variable	Intra-observer ICC value	Inter-observer ICC value
**LVEDV (mL)**	0.99	1.0
**LVESV (mL)**	0.99	0.94
**LVOT diameter (mm)**	0.98	0.93
**LVOT VTI (cm)**	0.99	1.0

ICC, intraclass correlation coefficient; LVEDV, left ventricular end-diastolic volume; LVESV, left ventricular end-systolic volume; LVOT, Left ventricular outflow tract; VTI, velocity–time integral
